# Burns complicating into *Klebsiella pneumoniae* pneumonia requiring ECMO therapy and with favourable outcome

**DOI:** 10.1093/jscr/rjag999

**Published:** 2026-11-04

**Authors:** Mustafa Suliman, Nashrah Umme, Amara Umme, Nissar Shaikh, Ibrahim Fawzy

**Affiliations:** Department of Medical Education, Hamad Medical Corporation, Doha, PO Box 3050, Qatar; Department of Medical Education, Deccan College of Medical Sciences, Hyderabad, PO Box 500058, India; Department of Medical Education, Deccan College of Medical Sciences, Hyderabad, PO Box 500058, India; Department of Anesthesia & ICU, Hamad Medical Corporation, Doha, PO Box 3050, Qatar; Department of Anesthesia & ICU, Hamad Medical Corporation, Doha, PO Box 3050, Qatar

**Keywords:** acute respiratory distress syndrome, burns, ECMO, *Klebsiella pneumoniae*, resistant hypoxia, tension pneumothorax

## Abstract

Burn injuries are common and carry high morbidity and mortality. Up to 40% of burn patients develop acute respiratory distress syndrome (ARDS) due to inhalational injury or infective pneumonia, and hypoxia may fail to respond to conventional therapy and ventilatory management. In this life-threatening scenario, extracorporeal membrane oxygenation (ECMO) therapy can be lifesaving, though literature on its use in burn patients with ARDS is limited. We report a case of *Klebsiella pneumoniae* pneumonia complicating into ARDS in a burn patient with resistant hypoxia, requiring ECMO therapy, with a favourable outcome.

## Introduction

The use of extracorporeal membrane oxygenation (ECMO) in burn patients with acute respiratory distress syndrome (ARDS) and resistant hypoxia is rare [1, 2]. In burn patients with ARDS resistant to traditional therapy, ECMO can serve as rescue therapy and be lifesaving. Although ECMO carries risks of bleeding and infection in burn patients, it remains viable for severe ARDS not responding to lung-protective ventilation and positional therapy [3]. Besides improving oxygenation and carbon dioxide removal, ECMO may help burn patients by reducing the need for ongoing fluid resuscitation and frequent surgical intervention, both difficult to manage alongside lung-protective ventilation [3]. We report a case of severe ARDS due to *Klebsiella pneumoniae* pneumonia with resistant hypoxia requiring ECMO therapy, with a favourable outcome.

## Case report

A 34-year-old man sustained facial, neck, upper back, and upper and lower extremity burns after a motor oil fire and explosion. He received intravenous fluids and multimodal analgesia before transfer to our hospital. On arrival he was awake, tachycardic (120–140 beats/minute) and tachypnoeic (30–40 breaths/minute), with stable blood pressure. He was intubated and ventilated, sedated with propofol, ketamine, and remifentanil, requiring noradrenaline to maintain haemodynamics. Oxygenation was adequate (98%–100%) with low ventilatory settings, and he was transferred to the surgical intensive care unit.

His upper-extremity burns were full-thickness with compartmental tension, requiring escharotomy (Fig. 1). Bronchoscopy showed no inhalational injury. Initial serum lactate was elevated (5 mmol/L) but normalized within 48 hours. He was started early on enteral high-protein feeds, which he tolerated well, and was weaned off vasopressors with minimal ventilatory settings by Day 4.

**Figure 1:**
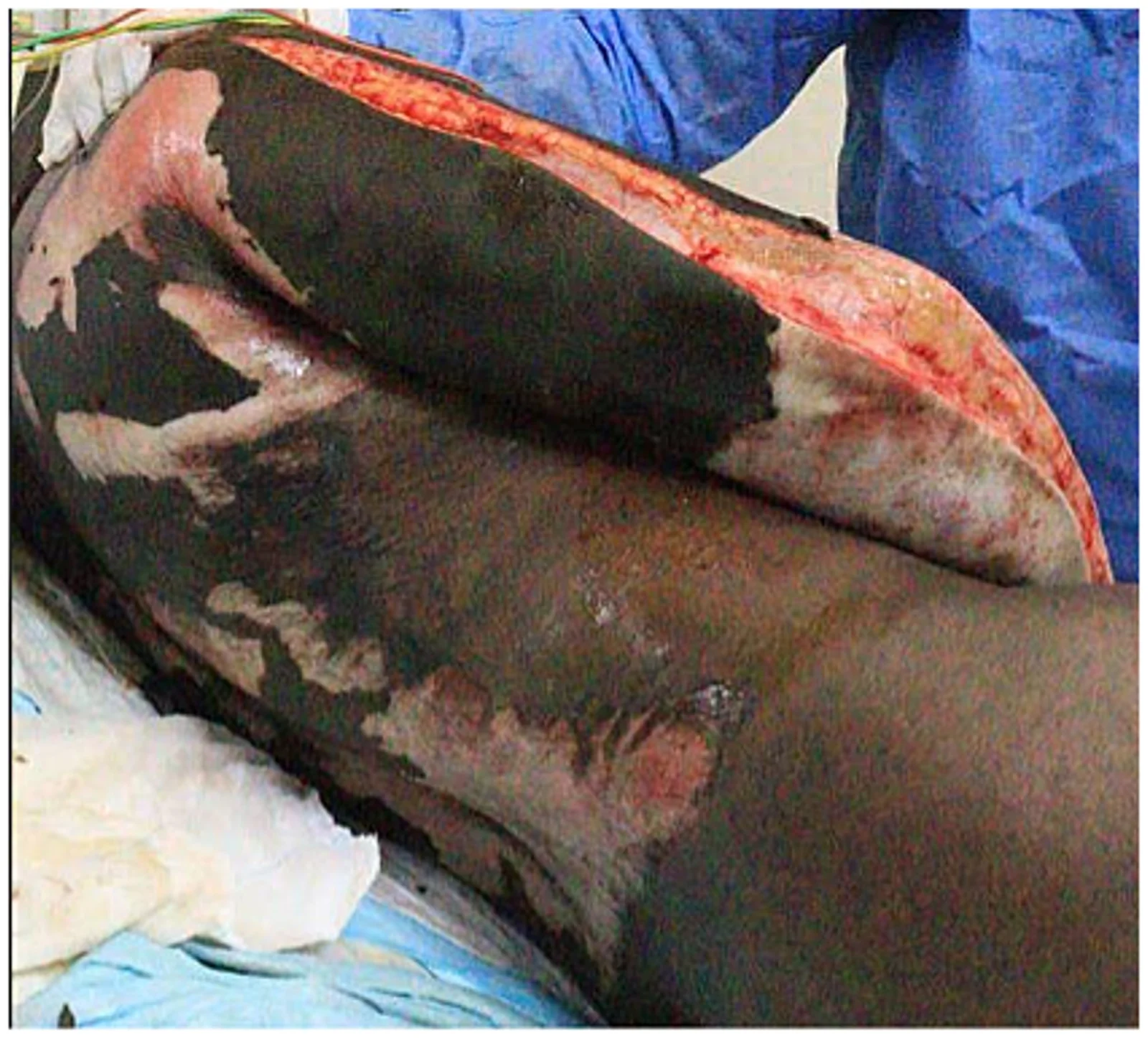
Clinical photograph of the upper limb showing a full-thickness burn with escharotomy performed to relieve compartmental tension.

On Day 7 he developed fever (39.1°C) with raised procalcitonin (8.5 ng/ml); cultures were obtained and piperacillin–tazobactam was started. Repeat bronchoscopy again showed no inhalational injury but revealed abundant tracheobronchial secretions. Chest radiography and computed tomography showed bilateral consolidation with ground-glass changes, and ventilatory requirements increased. Sputum culture grew *K. pneumoniae*, and antibiotics were escalated to meropenem.

By Day 11 he required maximal ventilatory support with 100% oxygen, and noradrenaline was restarted. He remained febrile, with chest radiography showing bilateral diffuse infiltrates (Fig. 2) and a partial pressure of oxygen to fraction of inspired oxygen (P/F) ratio of 134–147. Lung-protective ventilation was instituted, and he underwent three sessions of prone positioning. Despite this, the P/F ratio fell to 70–80, with high peak and plateau pressures and carbon dioxide retention. Given resistant hypoxia despite conventional ARDS management, the ECMO team was consulted and veno-venous ECMO was initiated, with an initial high sweep gas flow of 8–12 L/minute.

**Figure 2:**
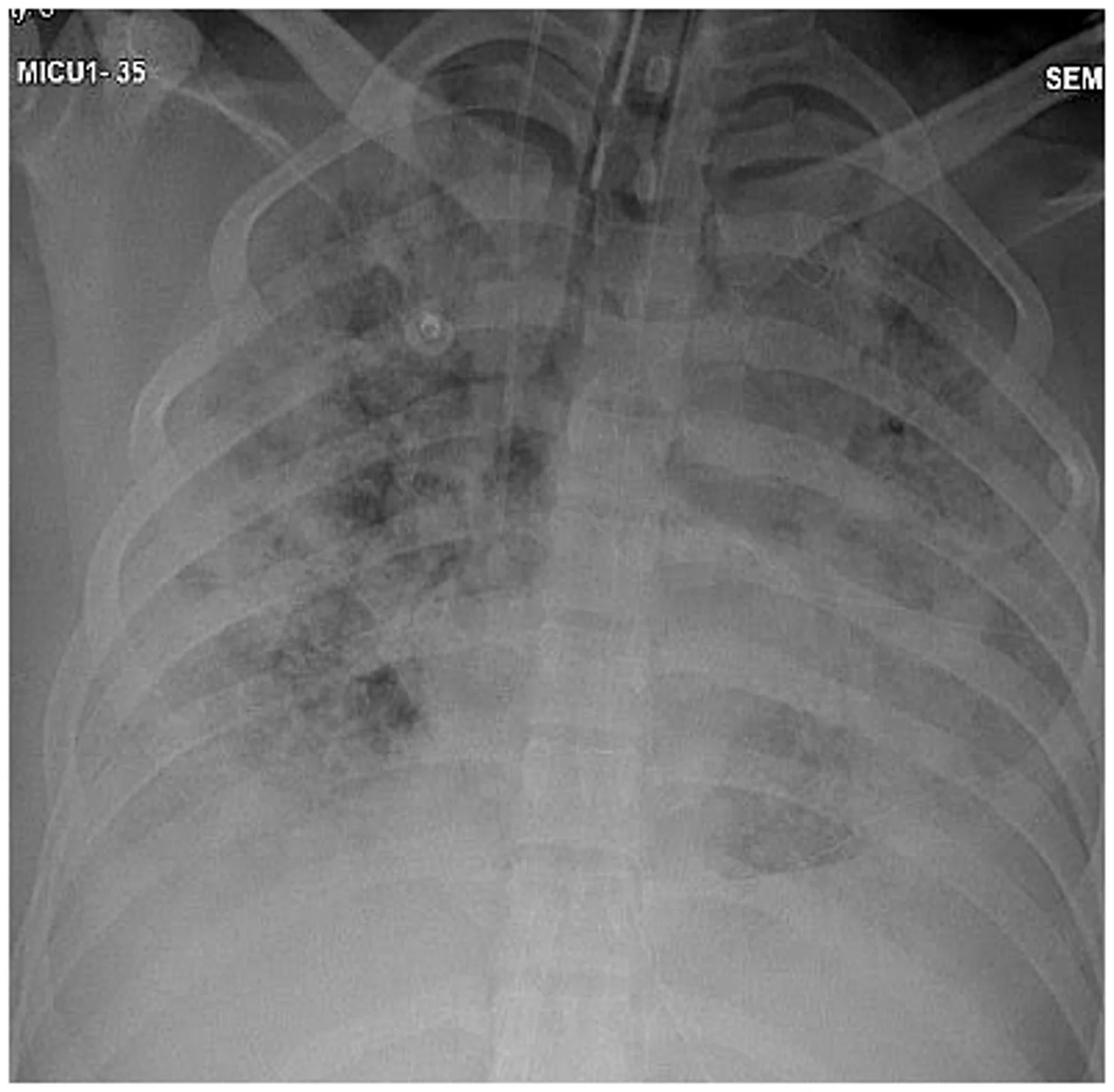
Anteroposterior chest radiograph on Day 11 showing bilateral diffuse pulmonary infiltrates consistent with acute respiratory distress syndrome.

He remained on ECMO for 17 days, during which he developed burn-wound infection with *K. pneumoniae* and *Candida albicans*, treated with meropenem, ciprofloxacin, and anidulafungin. Wound oozing necessitated multiple transfusions. On Day 21 he underwent bedside percutaneous tracheostomy. Weaning from ECMO began on Day 26, and he was decannulated on Day 30; lower-extremity venous Doppler was normal. Weaning from sedation, vasopressors and the ventilator began on Day 31.

On Day 32 he developed sudden desaturation with absent air entry. Point-of-care ultrasound and chest radiography (Fig. 3) revealed right tension pneumothorax, treated with chest tube insertion, with clinical improvement. He was weaned off sedation, vasopressors and the ventilator by Day 39, and underwent skin grafting of lower-limb burns on Days 52 and 56. His tracheostomy was decannulated on Day 61. He continued to improve, becoming awake and stable on an oral diet, and was transferred to the burns ward before discharge home on Day 73. He remains followed up in the outpatient burns clinic for minor reconstructive surgery and further skin grafting.

**Figure 3:**
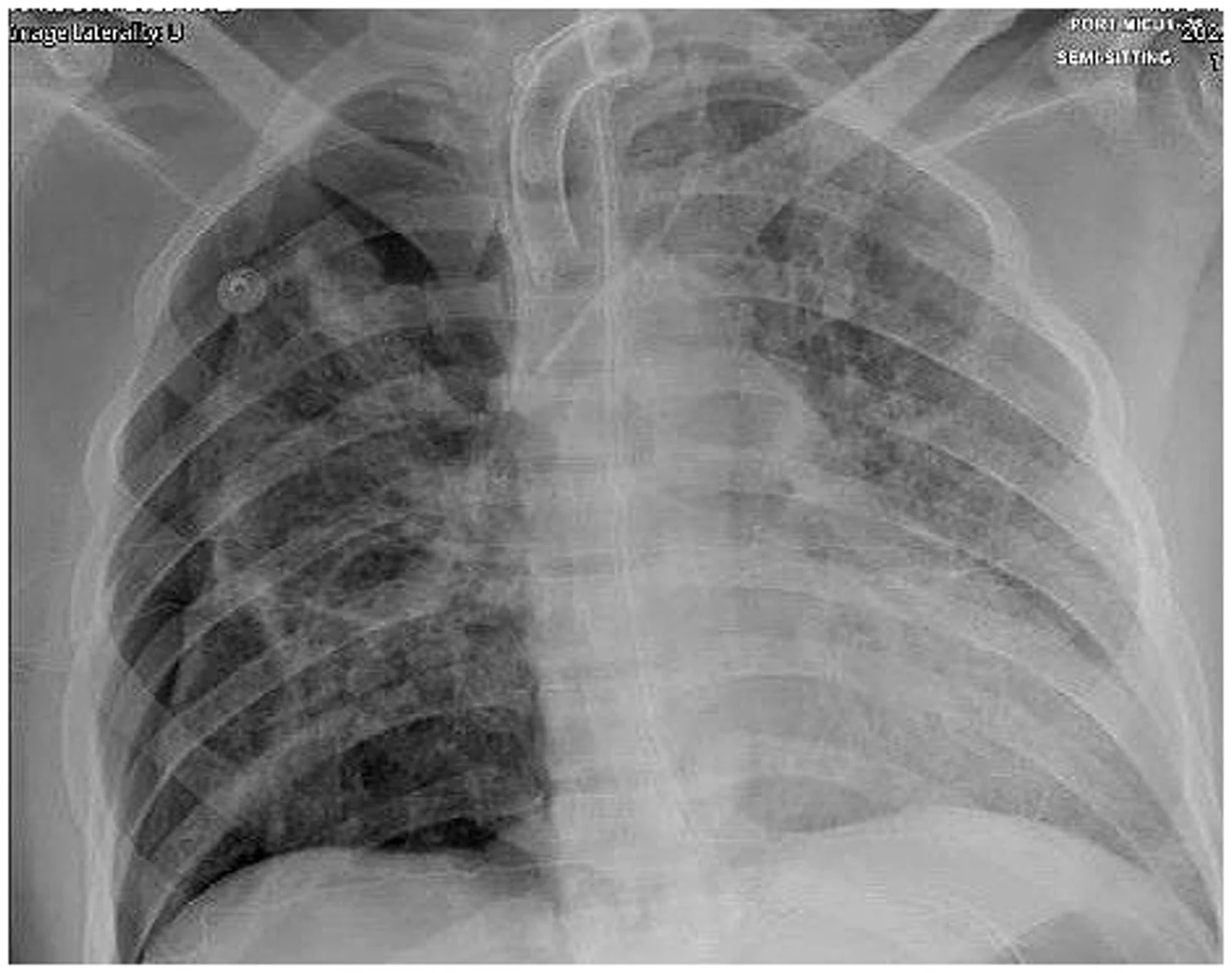
Anteroposterior chest radiograph on Day 32 showing right-sided tension pneumothorax during ventilator weaning.

## Discussion

ECMO supports patients with severe cardiac or respiratory failure of various aetiologies by oxygenating blood and removing carbon dioxide [4]. In severe ARDS with hypoxia resistant to traditional therapy and carbon dioxide retention, ECMO improves morbidity and mortality [5].

The use of ECMO in burn patients is rarely reported, owing to increased risks of bleeding and infection [1]. Inhalation injury and pulmonary infection are the leading causes of ARDS in burn patients [6]. In a large case series, infective pneumonia was the main cause of ARDS, with an average burn surface area of 37%; overall mortality in burn patients requiring ECMO was 43%, with acute kidney injury (AKI), hypercapnia, and low pH increasing mortality risk, and none of these patients received prone positioning before ECMO [7]. A second large study, from the US National Burn Registry, included 30 burn patients, 27% of whom had inhalation injury progressing to severe ARDS requiring ECMO, with mortality of 40%–50%; burn patients on ECMO had increased rates of AKI, sepsis, and longer ventilator and ICU days [8]. Fouché *et al*. [1], in their case series of burn patients requiring ECMO, reported no use of prone positioning before ECMO; most patients were young men, ECMO duration ranged from 2 to 29 days, with an average of 18 L of blood transfused, and mortality of 33%, with circuit thrombosis and cardiac arrest as causes of death.

Our patient was a young man with 40% burns and no inhalational injury who developed severe ARDS due to pneumonia, septic shock, and AKI. He received prone positioning before ECMO, was transfused 10.5 L of blood, and developed burn-wound infection during ECMO. He also developed pneumothorax during ventilator weaning, likely related to *K. pneumoniae* necrotizing pneumonia and variation in tidal volumes. Despite these complications, he had a favourable outcome. *K. pneumoniae* and *Staphylococcus aureus* are known causes of necrotizing pneumonia that create air-filled cavities (pneumatoceles) in the lung; these carry a high risk of rupture during weaning, resulting in tension pneumothorax [9]. Tension pneumothorax during ECMO reduces venous return and increases both ECMO duration and mortality in severe ARDS [10]. Although ECMO increases the risk of infection, AKI, and bleeding in burn patients, it remains lifesaving in severe ARDS with resistant hypoxia. Prone positioning can be used safely before ECMO, and early referral with prompt sepsis correction may improve outcomes.
